# Frequency of visits and examinations in the Public Dental Service in Finland – a retrospective analysis, 2001–2013

**DOI:** 10.1186/s12903-017-0436-8

**Published:** 2017-11-28

**Authors:** Jari Linden, Kim Josefsson, Eeva Widström

**Affiliations:** 1 Public Dental Service Lohja, Helsinki, Finland; 2 National Institute for Health and Welfare (THL), Helsinki, Finland; 3 Institute of Clinical Dentistry, Arctic University of Norway, Tromsø, Norway

**Keywords:** Register study, Examination intervals, Treatment need, Visiting interval, Public dental service

## Abstract

**Background:**

This study aimed to investigate longitudinally examination and visiting patterns in the Finnish Public Dental Service (PDS) and to relate these to patients’ treatment needs and international recommendations on examination intervals.

**Methods:**

Data on patients and their dental visits in the period 2001–2013 were collected from five municipal PDS-units serving a total population of 320,000 inhabitants and using the same database system. Ethical approval was given by the National Institute for Health and Welfare (THL) and permissions to use local data by the directors of health services in each unit. For each year, the numbers of visitors, those examined and those in need of basic periodontal or caries treatment (CPI >2 and D + d > 0) were calculated separately for young people (< 18 years), the working-aged (18–64 years) and the elderly (65+ years). Each individual’s examination and visiting intervals were counted. Multilevel modelling was used to study probabilities of being examined or in need of treatment and differences in examination and visiting intervals between groups and over time.

**Results:**

From 2001 to 2013, the number of visitors increased by 39.3% and the working-aged became the biggest patient group rather than the young. Compared with adults, the young were five times more likely to be examined (OR = 4.97) and three times less likely to require treatment (OR = 0.31). On average, 37% of the young, 73% of the working-aged and 63% of the elderly needed basic treatment. Multi-level analysis showed that the young had the shortest examination intervals and the working aged the longest (0.50 years longer). Most examination intervals of the young and the elderly were 1 year (65.2 - 77.0%), but only half (49.5%) of the working-aged were re-examined within 1 year. Over time, the examination intervals increased slightly in all groups. Most visiting intervals remained at 1 year.

**Conclusion:**

Young patients had mostly annual or biannual examinations, in line with recommendations. The examination intervals of working aged adults were considerably longer, and more of them needed treatment. The share of elderly among visitors remained low. The PDS seems to have access barriers for adults.

## Background

Dental and oral diseases affect all population groups and are difficult to recognise for lay persons. Thus, all people from young to old are advised to visit dentists (or dental hygienists) regularly. Besides ease of access, the use of dental services depends on financial and practical resources and cultural traditions. In the literature, the most commonly recommended revisiting period in dental care is 6 months [1]. There is little evidence supporting the recommendation, but dental professionals are convinced that frequent examinations allow disease to be detected and treated in time and preventive interventions to be delivered [2]. A clinical guideline in the UK recommends that the longest period between examinations for both children and adults should be 12 months; for adults maintaining good oral health and appropriate home care habits, this may be extended to 24 months [2]. The American and Irish Dental Associations recommend dental visits at least once a year to get a routine examination and cleaning [3, 4]. Commercial companies also recommend regular dental visits even for those who take excellent care of their teeth and gums at home – twice a year or more often, or just once a year [5].

In some countries the Medical Card dental schemes (Ireland) and public dental insurances reimburse (e.g. Sweden and Finland) an oral examination once a year.

In Finland, oral health care is delivered by the Public Dental Service (PDS) and private sector dentists. In 1972, the Primary Health Care Act obliged municipalities to organise and provide several types of primary health services to their inhabitants. In oral health care, the young were prioritised: annual examinations, all necessary care including preventive were to be offered free to all the young, everywhere in the country. Adults were to use private services and pay for them. Since the 1980s, adults were gradually given access to subsidised dental services in the PDS, starting with the youngest age groups. In parallel, the same adults were included in an evolving reimbursement scheme of private dental care by the National Health Insurance (NHI). Finally in 2002, those born before 1956 were also granted access to the PDS or to NHI-reimbursed private dental care [6].

Adults’ oral health has been monitored in three large national clinical (epidemiological) studies in 1980, 2000 and 2011 [7–9]. Rough statistics on the use of services, work force, costs and children’s oral health have been collected in the PDS since its start [10]. However, these data have not been very useful for evaluation or management in the PDS [11]. Along with improved data collection since the late 90’s, the performance of the public oral health care provision system can be studied more in detail. This is needed in the current tough economic and financial situation in Finland.

The aim of this study was to investigate examination intervals and dental visiting patterns in the PDS using register data in a longitudinal setting in 2001–2013, largely reflecting changes in coverage of adult services in the PDS. Additional aims were to study how examinations and treatment needs tallied, whether examination intervals changed during a longer period and how examination intervals accorded with recommendations in other countries.

## Methods

Five PDS units in Southern Finland that use a specific electronic patient registration system (WinHit), were asked to participate in the study. Ethical approval was given by the National Institute for Health and Welfare (THL) and permission to use the local data by the directors of health services in each PDS unit. In 2001, the number of inhabitants in these PDS units’ catchment areas was 271,301 and in 2013 320,055 (+15.2%) persons. The smallest unit had 25,679 and the biggest 182,072 inhabitants [12].

Using personal unique identifiers, the numbers of patients (*n* = 295,521), their dental visits (*n* = 3,281,300), examinations (*n* = 702,662), and treatment need in the years 2001–2013 were retrospectively collected from the municipal databases. Individual sequence numbers were created to replace all personal identifiers. For each year, the numbers of all patients that have at least one visit to the PDS during that year, those who have visited and been examined and among the examined, those in need of basic periodontal or caries treatment (CPI > 2, D + d > 0) were [13] calculated. Each individual’s visits and examinations were separately grouped by year and the time (in years) between visiting years and examination years was counted. All these numbers were counted separately for the young (< 18 years), the working-age adults (18–64 years) and the elderly (65+ years).

R 3.2 environment for statistical computing was used for data editing and descriptive analyses. The multi-level modelling procedures xtreg and xtlogit using Stata (StataCorp. 2013. Stata Statistical Software: Release 13. College Station, TX: StataCorp LP) were used to study the differences in the odds of being examined or being in need of treatment between age groups. Possible changes in these odds over time were also studied (Table 2). Multi-level modelling was also used to study the differences in examination intervals between the age groups, and possible changes in examination intervals over time (Table 4). We report both random effects and fixed effects coefficients. A random effects coefficient is a weighted average of the between and within individual factors; that is, it uses the variation between individuals as well as the variation within individuals over time. The fixed effects coefficient considers only the variation within individuals over time and indicates the magnitude of change in the outcome measure within the same individuals, provided that there are changes in the predictor variables.

## Results

From 2001 to 2013, the number of visitors to the PDS units increased by 39.3% (Table 1). Among the visitors, the proportion of children and adolescents decreased by 0.4%, the proportion of the working aged increased by 55.5% and that of the elderly by 506.1%. In 2001, the young made up the biggest patient group (51.5%), the working-aged the second biggest (45.7%) and the elderly the smallest (2.8%). In 2013, the working aged had become the biggest group (51.0%) followed by the young (36.8%) and the elderly (12.2%).

**Table 1:** Numbers of all patients (N) who visited the PDS, numbers (n) and proportions (%) (properly) examined and numbers (n) and proportions (%) of those examined who were in need of basic treatment by age group and year from 2001 to 2013 in five Finnish PDS units

	All patients having visited the PDS	Children and adolescents (<18 years)			Working-aged adults (18–64 years)			Elderly (65+ years)		
			Examined of the age group	In need of basic treatment of those examined		Examined of the age group	In need of basic treatment of those examined		Examined of the age group	In need of basic treatment of those examined
Year	N	n	n %	n %	n	n %	n %	n	n %	n %
2001	77,059	39,682	32,823 (82.7)	14,572 (44.4)	35,223	22,819 (64.8)	17,136 (75.1)	2154	1071 (49.7)	659 (61.5)
2002	77,160	39,297	32,604 (83.0)	14,120 (43.3)	35,429	21,234 (59.9)	15,799 (74.4)	2434	1184 (48.6)	764 (64.5)
2003	78,590	40,022	33,476 (83.6)	15,000 (44.8)	35,184	19,171 (54.5)	14,360 (74.9)	3384	1829 (54.0)	1225 (67.0)
2004	75,630	36,316	29,331 (80.8)	13,158 (44.9)	35,126	16,996 (48.4)	12,843 (75.6)	4188	1808 (43.2)	1167 (64.5)
2005	81,273	37,857	30,922 (81.7)	12,942 (41.9)	38,113	20,500 (53.8)	15,458 (75.4)	5303	2300 (43.4)	1499 (65.2)
2006	84,952	36,263	29,724 (82.0)	10,999 (37.0)	41,757	20,184 (48.3)	15,912 (78.8)	6932	2648 (38.2)	1889 (71.3)
2007	84,547	38,216	31,898 (83.5)	10,636 (33.3)	39,312	15,119 (38.5)	10,988 (72.7)	7019	2251 (32.1)	1425 (63.3)
2008	83,982	36,442	28,872 (79.2)	9536 (33.0)	40,277	17,613 (43.7)	13,121 (74.5)	7263	2810 (38.7)	1834 (65.3)
2009	95,821	40,746	32,018 (78.6)	9715 (30.3)	46,093	18,674 (40.5)	14,895 (79.8)	8982	3517 (39.2)	2530 (71.9)
2010	93,831	36,809	26,401 (71.7)	8611 (32.6)	47,468	19,721 (41.5)	13,033 (66.1)	9554	3932 (41.2)	2326 (59.2)
2011	99,047	40,433	31,321 (77.5)	10,111 (32.3)	48,093	19,754 (41.1)	12,967 (65.6)	10,521	4509 (42.9)	2557 (56.7)
2012	102,202	37,386	27,058 (72.4)	9515 (35.2)	52,674	22,611 (43.0)	15,552 (68.6)	12,142	5746 (47.3)	3144 (54.7)
2013	107,352	39,517	29,474 (74.6)	9794 (33.2)	54,779	22,082 (40.3)	15,225 (68.9)	13,056	6214 (47.6)	3402 (54.7)

The numbers and proportions of persons examined fluctuated during the study period (Table 1). Of all visitors during the 13 years, 80.7% had had at least one examination. Almost all (96.6%) of the young, 71.2%, of the working aged, and 63.2% of the elderly had been examined.

As Table 2 shows, compared with the elderly and the working aged, children had five times higher odds to be examined (OR = 4.97, 95% CI 4.89–5.05). The difference between the working aged and the elderly (OR = 1.09, 95% CI 1.08–1.12) was negligible. Among the young, the odds of being examined decreased slightly over time (OR = 0.75 years (9 months) per 5 years). A similar trend was seen among the working aged adults (OR = 0.69) but not among the elderly (OR = 1.04). The decreasing trend among the young was stronger (OR = 0.48 years (6 months) per 5 years) within individual analysis, e.g. when the same visiting individuals were followed repeatedly. A similar but much weaker trend could be detected among adults and the elderly.

**Table 2:** Odds ratios of being examined and being in need of treatment after an examination by age group. All patients included that have been examined at least once

	Random effects; number of at least once examined patients/all their visiting years	Fixed effects; number of patients examined at least two times/their pairs of examination years	Random effects; only visits with examinations/odds being in need of treatment	Fixed effects; including only patients examined at least two times/odds being in need of treatment
	n/observations OR (95% CI)	n/observations OR (95% CI)	n/observations OR (95% CI)	n/observations OR (95% CI)
All age groups	292700/1141446	151657/862118	235891/692269	85905/411062
< 18 years	4.97(4.89–5.05)		0.31(0.30–0.32)	
18–64 years	1.09(1.08–1.12)		1.72(1.67–1.77)	
65+ years	ref		ref	
Visiting year (per 5 years)	0.74(0.73–0.74)	0.60(0.59–0.60)	0.85(0.84–0.86)	1.52(1.77–2.09)
Only < 18 years	114116/498986	54161/345099	110254/395922	49646/263383
Visiting year (per 5 years)	0.75(0.74–0.75)	0.48(0.48–0.49)	0.96(0.95–0.97)	2.21(2.17–2.24)
Only 18–64 years	178423/549528	86367/411548	126868/256528	30309/108547
Visiting year (per 5 years)	0.69(0.69–0.70)	0.65(0.65–0.66)	0.83(0.82–0.84)	0.87(0.85–0.89)
Only 65+ years	32,139/92932	14,056/64912	20,281/39819	4532/15416
Visiting year (per 5 years)	1.04(1.02–1.06)	0.80(0.78–0.83)	0.76(0.73–0.79)	0.70(0.66–0.75)

All odds ratios are significant at *p* < 0.01

A smaller number of visitors (183 children, 6727 working aged adults and 1805 elderly) had not been examined although they had visited the PDS at least in three separate years.

Compared with the elderly, children had lower odds (OR = 0.31) and the working aged much higher odds (OR = 1.72) to be in need of treatment (Table 2). On average, 37.4% of the examined children and adolescents, 73.1% of the working aged and 63.1% of the elderly were considered to be in need of basic treatment. A weak decreasing trend in the odds of being in need for treatment was detected, in all three age groups during the study period. Fixed and random effects were similar among both adult groups. When the same children and adolescents were followed over time (fixed effects) an increasing trend towards need for treatment was observed (OR = 2.21), indicating that patients at risk were more often examined (Table 2).

Most examination intervals in the young and elderly (65.2–77.0%) were 1 year or less, but only half (49.5%) of those in the working age group had such a short interval. For them, examination intervals of three or more years were not unusual (21.2%; Table 3).

**Table 3:** Number, mean values and distribution (%) of the lengths of the visiting and examination intervals. Those persons included who had visits and examinations at least in 2 years

Number and length of the visiting interval					
Age group	n	Mean	1 year	2 years	3+ years
(years)		(years)	%	%	%
0–17	393,427	1.3	74.7	16.5	4.8
18–64	406,542	1.7	68.4	16.5	15.1
65+	70,429	1.4	79.0	11.6	9.4
Number and length of the examination interval					
Age group	n	Mean	1 year	2 years	3+ years
(years)		(years)	%	%	%
0–17	238,585	1.4	65.2	28.6	6.2
18–64	85,647	2.0	49.5	29.3	21.2
65+	13,152	1.4	77.0	15.9	7.1

Multilevel analysis (Table 4) confirmed that, on average, children had the shortest examination intervals (reference) and those of working age the longest intervals (0.5 years, 6 months longer). The elderly had an interval between these two groups (0.16 years, about 1.9 months longer than the young). Over time, the examination intervals increased slightly in all age groups. The largest trend, 0.16 years (1.9 months) per 5 years, was found for the children. When the same regularly visiting individuals were followed (fixed effects), the examination intervals increased more over time than when all patients were included (random effects). In all age groups, the change in visiting intervals over time was close to zero (Table 4).

**Table 4:** The effect of age group and visiting year on the visit and examination intervals

	Random effects, visit interval	Fixed effects, visit interval	Random effects, exam interval	Fixed effects, exam interval
	n/observations B(95% CI)	n/observations B(95% CI)	n/observations B(95% CI)	n/observations B(95% CI)
All age groups	190,811/657200	190,811/657200	128,329/337242	128,329/337242
< 18 years	ref		ref	
18–64 years	0.13(0.13–0.14)		0.50(0.49–0.51)	
65+ years	−0.03(−0.04−−0.03)		0.16(0.15–0.18)	
Visiting year (per 5 years)	0.02(0.01–0.02)	0.01(0.00–0.01)	0.13(0.12–0.13)	0.20(0.19–0.21)
Only < 18 years	78,560/289819	78,560/289819	72,812/214232	72,812/214232
Visiting year (per 5 years)	0.02(0.02–0.03)	-0.03(−0.04--0.03)	0.16(0.15–0.16)	0.19(0.19–0.20)
Only 18–64 years	109,527/307174	109,527/307174	55,607/103482	55,607/103482
Visiting year (per 5 years)	−0.02(−0.02--0.01)	0.01(0.01–0.02)	0.07(0.06–0.08)	0.15(0.13–0.16)
Only 65+ years	20,691/60207	20,691/60207	10,001/19528	10,001/19528
Visiting year (per 5 years)	0.05(0.03–0.06)	0.05(0.04–0.07)	0.07(0.05–0.10)	0.20(0.16–0.24)

Patients with at least two visits or examinations included. Second visit in 2005 or later. Only observations with an interval of 4 or less years included

Visiting year is a continuous and age group a categorical predictor

All coefficients significant at *p* < 0.01 level

B = change in years per one unit change in the predictor

## Discussion

Five PDS units using a special patient database system were chosen for this study. The data were collected from each PDS unit’s database backup by the same expert using the same script. Recording of data is mandatory and in addition, part of each dentist’s salary is based on treatment measures provided. The quality of data from the PDS records has been considered reliable [14]. Unfortunately, only simple clinical indicators of treatment need (D + d and CPI) were available. Thus, the treatment needs are likely to be underestimated, especially in adults.

During the 13-year period, the population in the five PDS units’ up-take areas increased by 15.2%; the young by 9.6%, the working aged by 12.1% and the elderly by 31.6% [12]. Throughout the study period, the annual coverage (proportion of the population that attended the PDS) of children and adolescents was high, around 75%. The coverage of working aged adults increased from 20 to 28% and that of the elderly from 5 to 22%. These figures are similar to national figures and indicate that the selected PDS-units are not outliers among the Finnish PDS units [10].

During the study period, the PDS changed from being an oral health care provider mainly for the young to be a care provider for the whole population. This was the intention with the Oral Health Care Reform in 2001–2002 [15]. High numbers of older adults, often with accumulated treatment needs, came to the PDS, partly due to low patient fees [15]. This resulted in long waiting lists because the PDS had received few new resources but had a legal obligation to organise emergency dental services for all inhabitants in its uptake-area [16]. This study showed that although the older population grew dramatically, their share as patients in the PDS grew only from three to 12% in spite of the “free access”. One explanation can be that edentulousness has been and still is common in this group; in 2000, 44% of all the 65+ year olds and, in 2011, 17% of the 65–74 year age group and 40% of the 75+ year olds had lost all their teeth [8, 9]. On the other hand, when access to the PDS was restricted to the young, most dentate adults had to use private services and many of them probably have continued to visit their “own” dentists.

It was obvious from our results that the young were taken care of differently from the adults. They were five times more likely to be examined although they were three times less likely to be in need of treatment. Also the examination intervals for working aged adults were on average 6 months longer and, for the elderly, 2 months longer than for the young. Furthermore, while almost all the young (97%) had been examined, the corresponding figures for working aged adults (71%) and for the elderly (63%) were much lower. The young (< 18 years) have for several decades been heavily prioritised in the PDS and all care including orthodontics has been free of charge. The PDS is popular and only 1% of the young use private services [17].

The results of this study indicate that the personnel resources in the PDS are not sufficient or are inefficiently used as regards adult care. In order to transfer clinical activities from the young towards adults known to have greater treatment needs, there have been attempts to individualise and extend patients’ recall intervals, starting with the children and younger adults [18]. This “thinning-out the examinations” philosophy has later been applied to other adults, too. Thus, for example, the PDS in Helsinki recommends that a dentist should examine “healthy adults” in every 4 to 5 years and visit a hygienist every 2 to 3 years [19]. However, most of the “new” patients (born before 1956) who came to the PDS in 2002 and after were not likely to have been healthy adults. The national studies in 2000 and 2011 revealed great treatment needs in the adult population, such as missing teeth needing prosthetic replacements, caries and broken fillings and not least periodontal disease of various degrees and generally bad oral hygiene especially in the low income and low education groups [8, 9]. A recent study in the PDS of a bigger city showed that there were high numbers of middle aged and older “heavy consumers” with low socioeconomic background showing up repeatedly for lost fillings and other semi-acute treatment and they were not offered examinations nor regular treatment [20, 21]. Most PDS units recall the young only, in contrast to the private sector, which uses recall as one of its most important marketing tools [22]. In the PDS, adults are advised to make new appointments after a given time period and are then put on the waiting list again [23]. This happens partly due to the Care Guarantee legislation from 2005, which stipulates that emergency services have to be given within 3 days and non-urgent care has to be started within 6 months for all those asking for treatment in the PDS. Postponing further treatment sessions and old (adult) patients’ new treatment episodes has become a way to cope with the legal requirements. It has also been suggested that many PDS dentists used to treating younger patients do not feel competent to treat older adults with more complex treatment needs [24] and our study indicates that older adults probably still have “invisible” access barriers in the PDS. Thus, the findings reflect national special features in the care provision system, such as contradictory incentives between public and private care providers in adult dental care, the lack of national steering and vague local leadership [25]. As a result, the PDS continues to prioritise the young and is not successful in meeting the internationally recommended examination intervals for adults. This might be one reason for the persisting oral health inequalities in Finland. During the current economic recession, acquiring resources for public oral health care is challenging. As an alternative, the PDS should probably reorganise its treatment processes, review the division of labour and the remuneration system and put more effort into improving adult patients’ oral hygiene and home care and into continuing education for its own staff.

## Conclusions

Most young patients in the PDS are recalled regularly and in line with best practice guidelines. Due to lack of a recall system, adults’ examination intervals had increased and, in spite of much greater treatment needs, were longer than the recommended in other countries. However, visiting intervals for all age groups were shorter, around 1 year and they did not increase. This suggests that the examination intervals for adults are too long, especially in relation to their treatment needs.
